# Dichloridobis[3-(4-meth­oxy­phen­yl)-2-methyl-5-(piperidin-1-yl)-2,3-di­hydro-1,2,4-oxa­diazole-κ*N* ^4^]platinum(II)

**DOI:** 10.1107/S1600536813018059

**Published:** 2013-07-06

**Authors:** Andreii S. Kritchenkov, Leonid V. Lavnevich, Galina L. Starova, Nadezhda A. Bokach, Valentina A. Kalibabchuk

**Affiliations:** a Department of Chemistry, Saint Petersburg State University, Universitetsky Pr. 26, 198504 Stary Petergof, Russian Federation; b O.O. Bohomolets National Medical University, Department of General Chemistry, Shevchenko blvd. 13, 01004 Kiev, Ukraine

## Abstract

In title compound, [PtCl_2_(C_15_H_21_N_3_O_2_)_2_], the Pt^II^ cation, located on an inversion center, is coordinated by two Cl^−^ anions and two 3-(4-meth­oxy­phen­yl)-2-methyl-5-(piperidin-1-yl)-2,3-di­hydro-1,2,4-oxa­diazole ligands in a distorted Cl_2_N_2_ square-planar geometry. The di­hydro­oxa­diazole and piperidine rings display envelope (with the non-coordinating N atom as the flap atom) and chair conformations, respectively. In the crystal, weak C—H⋯Cl hydrogen bonds link the mol­ecules into supra­molecular chains running along the *b* axis. The piperidine ring is disordered over two positions with the occupancy ratio of 0.528 (4):0.472 (4).

## Related literature

For applications of platinum species bearing N-bound 2,3-di­hydro-1,2,4-oxa­diazo­les, see: Coley *et al.* (2008 ▶); Wagner *et al.* (2010 ▶). For the synthesis of platinum complexes bearing 2,3-di­hydro-1,2,4-oxa­diazole ligands, see: Kritchenkov *et al.* (2011 ▶). For related structures, see: Bokach & Kukushkin (2006 ▶); Bokach *et al.* (2011 ▶); Fritsky *et al.* (2006 ▶); Penkova *et al.* (2009 ▶). For standard bond lengths, see: Allen *et al.* (1987 ▶).
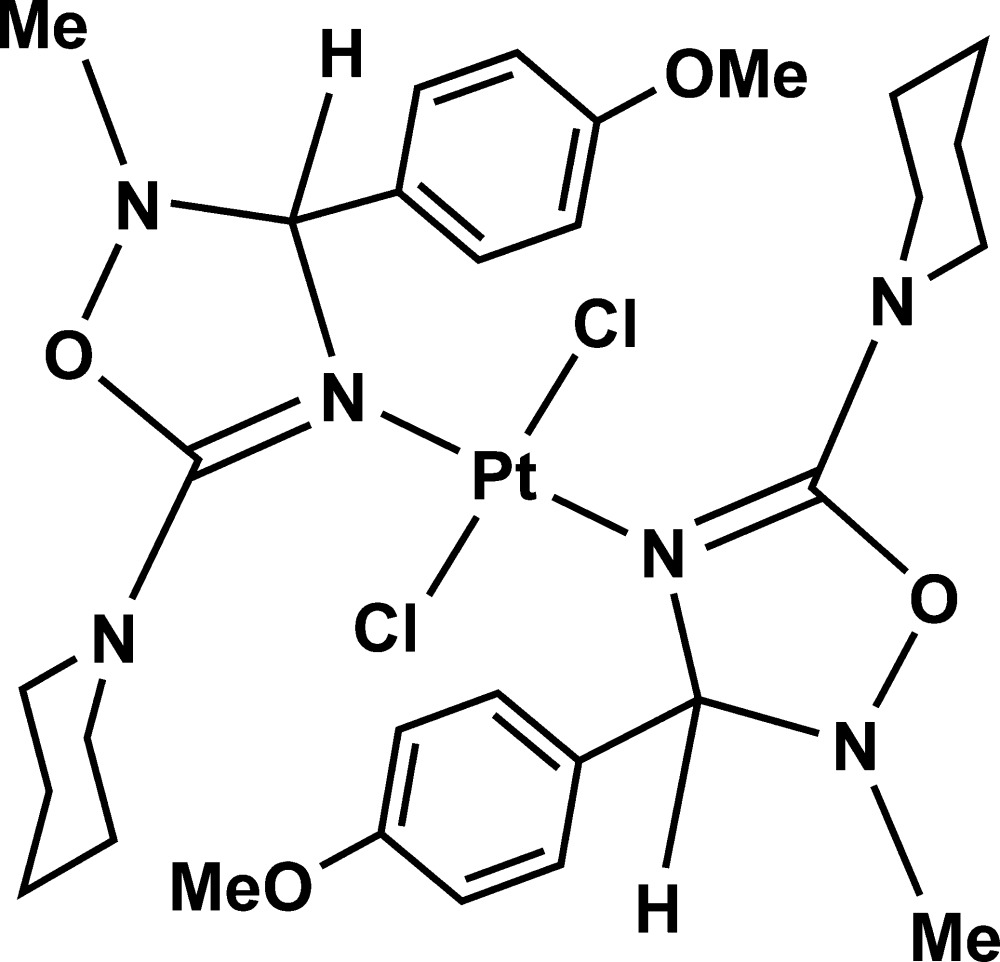


## Experimental

### 

#### Crystal data

[PtCl_2_(C_15_H_21_N_3_O_2_)_2_]
*M* *_r_* = 816.69
Monoclinic, 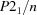

*a* = 12.77795 (19) Å
*b* = 8.57581 (15) Å
*c* = 15.1086 (3) Å
β = 95.0717 (17)°
*V* = 1649.13 (5) Å^3^
*Z* = 2
Mo *K*α radiation
μ = 4.46 mm^−1^
*T* = 100 K
0.22 × 0.18 × 0.15 mm

#### Data collection

Agilent Xcalibur Eos diffractometer
Absorption correction: multi-scan (*DENZO*/*SCALEPACK*; Otwinowski & Minor, 1997 ▶) *T*_min_ = 0.617, *T*_max_ = 1.000
13705 measured reflections
5072 independent reflections
3997 reflections with *I* > 2σ(*I*)
*R*_int_ = 0.026

#### Refinement

*R*[*F* ^2^ > 2σ(*F* ^2^)] = 0.020
*wR*(*F* ^2^) = 0.042
*S* = 1.05
5072 reflections
253 parameters
H-atom parameters constrained
Δρ_max_ = 1.00 e Å^−3^
Δρ_min_ = −0.64 e Å^−3^

### 

Data collection: *CrysAlis PRO* (Agilent, 2012 ▶); cell refinement: *CrysAlis PRO*; data reduction: *CrysAlis PRO*; program(s) used to solve structure: *SIR2004* (Burla *et al.*, 2005 ▶); program(s) used to refine structure: *SHELXL97* (Sheldrick, 2008 ▶); molecular graphics: *DIAMOND* (Brandenburg, 2009 ▶); software used to prepare material for publication: *SHELXL97*.

## Supplementary Material

Crystal structure: contains datablock(s) I, global. DOI: 10.1107/S1600536813018059/xu5715sup1.cif

Structure factors: contains datablock(s) I. DOI: 10.1107/S1600536813018059/xu5715Isup2.hkl

Additional supplementary materials:  crystallographic information; 3D view; checkCIF report

## Figures and Tables

**Table 1:** Selected bond lengths (Å)

Pt1—N3	2.0293 (16)
Pt1—Cl1	2.3108 (5)

**Table 2:** Hydrogen-bond geometry (Å, °)

*D*—H⋯*A*	*D*—H	H⋯*A*	*D*⋯*A*	*D*—H⋯*A*
C14—H14*C*⋯Cl1^i^	0.96	2.76	3.423 (3)	127

Symmetry code: (i) 
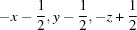
.

**Fig. 1.**
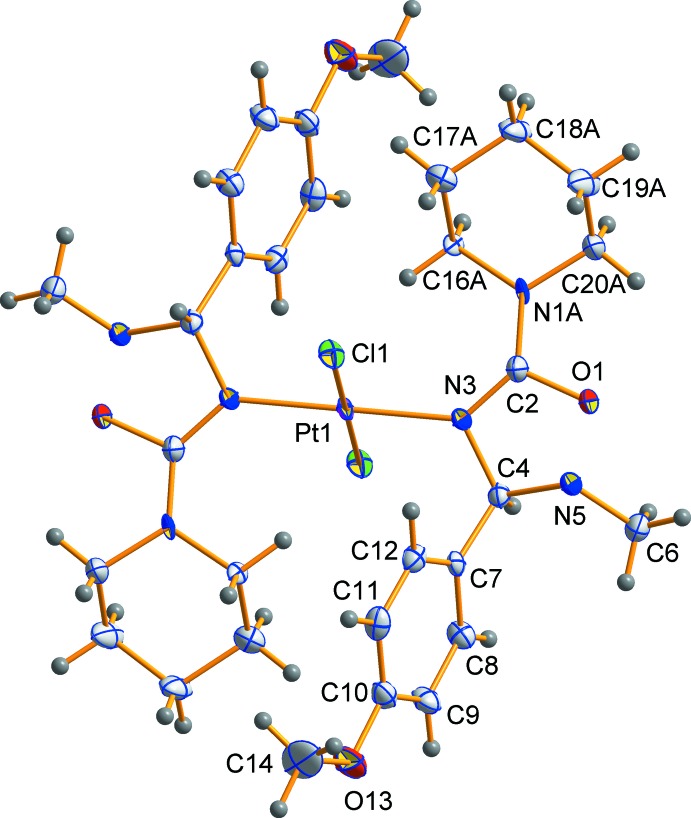
View to the C30H42Cl2N6O4Pt complex in the structure of 1. Thermal ellipsoids are drawn at the 50% probability level.

## supplementary crystallographic information

### Comment

In the past decade, a great attention has been paid to metal-mediated cycloaddition (CA) of dipoles to nitriles because the activation of nitrile substrates by a suitable metal center often results in promotion of CAs, which are not feasible in so-called pure organic chemistry (Coley *et al.*, 2008, Wagner *et al.*, 2010). Thus, the metal-mediated CA represents an efficient route to free and/or coordinated heterocycles that could be either difficult to obtain or even inaccessible *via* metal-free protocols (Bokach *et al.*, 2006, 2011). Furthermore, an interest in 2,3-dihydro-1,2,4-oxadiazole and their platinum complexes is caused by their potential application in medicine. Despite 2,3-dihydro-1,2,4-oxadiazoles are known, examples of 5-dialkylamino-2,3-dihydro-1,2,4-oxadiazoles and, in particular, their metal complexes are rare. Therefore, the synthesis of new complexes bearing the rare heterocycles as ligands and investigation of their properties, including their biological activity, are of interest. As an amplification of our project focused on metal-mediated CA and reactivity of metal-bound dialkylcyanamides (Kritchenkov *et al.*, 2011)) we synthesized and characterized the title compound by a single-crystal X-ray diffraction.

In the crystal structure of the title compound, the Pt atom is in the inversion center and coordinated by two Cl atoms and two N atoms (Fig. 1) of the heterocycles in *trans*-position. The Pt(1)–N(3) bond length (Table 1) is typical for (imine)Pt^II^ species (Allen *et al.*, 1987). The N(3)–C(2) (1.307 (3) Å) distance is characteristic for the N=C double bond (Fritsky *et al.*, 2006; Penkova *et al.*, 2009), while the N(3)–C(4) and N(5)–C(4) (1.472 (3) and 1.480 (3) Å), correspondingly, are specific for the N–C single bonds (Allen *et al.*, 1987). In the complex, the C(4) atom of the heterocyclic ligand exhibits the *RR*/*SS* configuration.

In the crystal structure, the complexes interact with each other *via* the weak C—H···Cl hydrogen bond (Table 2), forming the supramolecular chains running along the b axis.

### Experimental

Title compound was synthesized and isolated as pure solid by the described method (Kritchenkov *et al.*, 2011). The crystal was obtained by a slow evaporation of chloroform (RT) solution of the title compound. Hexane was added to the solution for improvement of crystallization of the complex.

### Refinement

The piperidine ring was found to be disordered over two positions, the occupancies were refined to 0.528 (4)/0.472 (4). H atoms were placed in calculated positions with C—H = 0.93–0.98 Å and included in the refinement in the riding model approximation, U_iso_(H) set to 1.5U_eq_(C) for methyl H atoms and 1.2U_eq_(C) for the others.

### Crystal data

**Table:**

[PtCl_2_(C_15_H_21_N_3_O_2_)_2_]	*F*(000) = 816
*M**_r_* = 816.69	*D*_x_ = 1.645 Mg m^−^^3^
Monoclinic, *P*2_1_/*n*	Mo *K*α radiation, λ = 0.71073 Å
Hall symbol: -P 2yn	Cell parameters from 6284 reflections
*a* = 12.77795 (19) Å	θ = 2.7–31.7°
*b* = 8.57581 (15) Å	µ = 4.46 mm^−^^1^
*c* = 15.1086 (3) Å	*T* = 100 K
β = 95.0717 (17)°	Prizm, light-yellow
*V* = 1649.13 (5) Å^3^	0.22 × 0.18 × 0.15 mm
*Z* = 2	

### Data collection

**Table:**

Agilent Xcalibur Eos diffractometer	5072 independent reflections
Radiation source: Enhance (Mo) X-ray Source	3997 reflections with *I* > 2σ(*I*)
Graphite monochromator	*R*_int_ = 0.026
Detector resolution: 16.2096 pixels mm^-1^	θ_max_ = 31.8°, θ_min_ = 2.7°
ω scans	*h* = −18→17
Absorption correction: multi-scan (*DENZO*/*SCALEPACK*; Otwinowski & Minor, 1997)	*k* = −11→11
*T*_min_ = 0.617, *T*_max_ = 1.000	*l* = −21→21
13705 measured reflections	

### Refinement

**Table:**

Refinement on *F*^2^	Primary atom site location: structure-invariant direct methods
Least-squares matrix: full	Secondary atom site location: difference Fourier map
*R*[*F*^2^ > 2σ(*F*^2^)] = 0.020	Hydrogen site location: inferred from neighbouring sites
*wR*(*F*^2^) = 0.042	H-atom parameters constrained
*S* = 1.05	*w* = 1/[σ^2^(*F*_o_^2^) + (0.0128*P*)^2^] where *P* = (*F*_o_^2^ + 2*F*_c_^2^)/3
5072 reflections	(Δ/σ)_max_ = 0.001
253 parameters	Δρ_max_ = 1.00 e Å^−^^3^
0 restraints	Δρ_min_ = −0.64 e Å^−^^3^

### Special details

**Table:**

Experimental. The piperidine ring was found to be disordered over two positions with the occupancies 0.528/0.472. The non-hydrogen atoms were refined anisotropically. Carbon- and nitrogen-bonded H atoms were placed in calculated positions and were included in the refinement in the "riding" model approximation, with U_iso_(H) set to 1.5U_eq_(C) and C—H 0.96 Å for the methyl groups, 1.2U_eq_(C) and C—H 0.98 Å for the tertiary CH groups, 1.2U_eq_(C) and C—H 0.93 Å for the carbon atoms of the benzene rings, and 1.2Ueq(N) and N—H 0.91 Å for the tertiary NH groups.
Geometry. All esds (except the esd in the dihedral angle between two l.s. planes) are estimated using the full covariance matrix. The cell esds are taken into account individually in the estimation of esds in distances, angles and torsion angles; correlations between esds in cell parameters are only used when they are defined by crystal symmetry. An approximate (isotropic) treatment of cell esds is used for estimating esds involving l.s. planes.
Refinement. Refinement of F^2^ against ALL reflections. The weighted R-factor wR and goodness of fit S are based on F^2^, conventional R-factors R are based on F, with F set to zero for negative F^2^. The threshold expression of F^2^ > 2sigma(F^2^) is used only for calculating R-factors(gt) etc. and is not relevant to the choice of reflections for refinement. R-factors based on F^2^ are statistically about twice as large as those based on F, and R- factors based on ALL data will be even larger.

### Fractional atomic coordinates and isotropic or equivalent isotropic displacement parameters (Å^2^)

**Table:**

	*x*	*y*	*z*	*U*_iso_*/*U*_eq_	Occ. (<1)
Pt1	0.0000	0.5000	0.0000	0.01251 (3)	
Cl1	−0.01009 (4)	0.73071 (6)	0.07781 (3)	0.02001 (11)	
N3	0.13951 (13)	0.4505 (2)	0.06907 (11)	0.0160 (4)	
N5	0.24273 (14)	0.3416 (2)	0.18442 (12)	0.0217 (4)	
O1	0.30566 (12)	0.4436 (2)	0.12999 (11)	0.0321 (4)	
O13	−0.17565 (13)	0.0800 (2)	0.31245 (12)	0.0383 (4)	
C2	0.23174 (17)	0.5175 (3)	0.07460 (16)	0.0246 (5)	
C4	0.15295 (15)	0.3037 (2)	0.11921 (13)	0.0177 (4)	
H4	0.1726	0.2199	0.0798	0.021*	
C6	0.31081 (18)	0.2079 (3)	0.20773 (16)	0.0315 (6)	
H6A	0.2708	0.1275	0.2334	0.047*	
H6B	0.3674	0.2397	0.2499	0.047*	
H6C	0.3389	0.1687	0.1552	0.047*	
C7	0.06220 (15)	0.2522 (2)	0.16910 (13)	0.0164 (4)	
C8	0.04130 (17)	0.0942 (3)	0.17501 (14)	0.0215 (5)	
H8	0.0805	0.0232	0.1451	0.026*	
C9	−0.03721 (18)	0.0406 (3)	0.22485 (16)	0.0251 (5)	
H9	−0.0492	−0.0659	0.2295	0.030*	
C10	−0.09774 (17)	0.1454 (3)	0.26766 (14)	0.0239 (5)	
C11	−0.07848 (17)	0.3051 (3)	0.26166 (13)	0.0229 (5)	
H11	−0.1193	0.3761	0.2899	0.027*	
C12	0.00224 (17)	0.3571 (3)	0.21309 (13)	0.0201 (5)	
H12	0.0162	0.4633	0.2100	0.024*	
C14	−0.2437 (2)	0.1821 (4)	0.3538 (2)	0.0573 (9)	
H14A	−0.2750	0.2542	0.3106	0.086*	
H14B	−0.2043	0.2386	0.4004	0.086*	
H14C	−0.2978	0.1226	0.3783	0.086*	
N1A	0.2753 (9)	0.6289 (16)	0.0262 (9)	0.024 (2)	0.528 (4)
C16A	0.2130 (12)	0.7065 (14)	−0.0497 (9)	0.0144 (16)	0.528 (4)
H16A	0.1405	0.6719	−0.0531	0.017*	0.528 (4)
H16B	0.2417	0.6808	−0.1052	0.017*	0.528 (4)
C17A	0.2194 (3)	0.8876 (5)	−0.0328 (3)	0.0284 (12)	0.528 (4)
H17A	0.1838	0.9418	−0.0832	0.034*	0.528 (4)
H17B	0.1837	0.9130	0.0194	0.034*	0.528 (4)
C18A	0.3334 (4)	0.9427 (6)	−0.0193 (3)	0.0326 (13)	0.528 (4)
H18A	0.3667	0.9297	−0.0740	0.039*	0.528 (4)
H18B	0.3353	1.0526	−0.0040	0.039*	0.528 (4)
C19A	0.3931 (5)	0.8491 (7)	0.0547 (4)	0.0465 (18)	0.528 (4)
H19A	0.4664	0.8797	0.0595	0.056*	0.528 (4)
H19B	0.3650	0.8729	0.1107	0.056*	0.528 (4)
C20A	0.3849 (4)	0.6741 (7)	0.0376 (4)	0.0412 (16)	0.528 (4)
H20A	0.4188	0.6479	−0.0154	0.049*	0.528 (4)
H20B	0.4202	0.6178	0.0873	0.049*	0.528 (4)
N1B	0.2575 (10)	0.6571 (17)	0.0444 (10)	0.0193 (18)	0.472 (4)
C16B	0.2104 (14)	0.7419 (16)	−0.0325 (10)	0.022 (2)	0.472 (4)
H16C	0.1836	0.8409	−0.0133	0.026*	0.472 (4)
H16D	0.1518	0.6825	−0.0602	0.026*	0.472 (4)
C17B	0.2883 (4)	0.7704 (7)	−0.0991 (3)	0.0319 (14)	0.472 (4)
H17C	0.2550	0.8296	−0.1485	0.038*	0.472 (4)
H17D	0.3109	0.6714	−0.1219	0.038*	0.472 (4)
C18B	0.3841 (4)	0.8600 (7)	−0.0579 (4)	0.0354 (15)	0.472 (4)
H18C	0.3632	0.9635	−0.0406	0.043*	0.472 (4)
H18D	0.4355	0.8707	−0.1010	0.043*	0.472 (4)
C19B	0.4330 (5)	0.7707 (9)	0.0244 (4)	0.0401 (17)	0.472 (4)
H19C	0.4602	0.6712	0.0062	0.048*	0.472 (4)
H19D	0.4910	0.8302	0.0531	0.048*	0.472 (4)
C20B	0.3518 (4)	0.7444 (7)	0.0884 (4)	0.0313 (15)	0.472 (4)
H20C	0.3290	0.8440	0.1102	0.038*	0.472 (4)
H20D	0.3823	0.6848	0.1388	0.038*	0.472 (4)

### Atomic displacement parameters (Å^2^)

**Table:**

	*U*^11^	*U*^22^	*U*^33^	*U*^12^	*U*^13^	*U*^23^
Pt1	0.00901 (5)	0.01308 (6)	0.01504 (5)	−0.00236 (4)	−0.00124 (3)	−0.00214 (5)
Cl1	0.0188 (2)	0.0172 (3)	0.0235 (2)	−0.00130 (19)	−0.00079 (19)	−0.0069 (2)
N3	0.0136 (8)	0.0151 (8)	0.0185 (9)	−0.0028 (6)	−0.0025 (7)	0.0011 (7)
N5	0.0157 (9)	0.0199 (10)	0.0283 (10)	−0.0044 (7)	−0.0048 (8)	0.0064 (8)
O1	0.0148 (8)	0.0358 (9)	0.0435 (10)	−0.0079 (7)	−0.0099 (7)	0.0215 (8)
O13	0.0265 (10)	0.0429 (11)	0.0482 (11)	−0.0006 (8)	0.0184 (8)	0.0072 (9)
C2	0.0170 (10)	0.0261 (13)	0.0292 (12)	−0.0034 (9)	−0.0059 (9)	0.0084 (10)
C4	0.0147 (10)	0.0155 (11)	0.0223 (11)	0.0003 (8)	−0.0016 (8)	−0.0010 (8)
C6	0.0219 (12)	0.0269 (13)	0.0441 (15)	0.0002 (10)	−0.0059 (11)	0.0114 (11)
C7	0.0116 (9)	0.0200 (11)	0.0166 (10)	−0.0031 (8)	−0.0033 (7)	0.0008 (8)
C8	0.0200 (11)	0.0199 (12)	0.0249 (11)	−0.0012 (9)	0.0038 (9)	−0.0053 (9)
C9	0.0225 (12)	0.0213 (12)	0.0318 (13)	−0.0069 (9)	0.0037 (10)	−0.0019 (9)
C10	0.0182 (11)	0.0328 (14)	0.0207 (11)	−0.0034 (9)	0.0028 (9)	0.0021 (10)
C11	0.0215 (11)	0.0296 (13)	0.0176 (10)	0.0065 (9)	0.0023 (9)	−0.0039 (9)
C12	0.0229 (11)	0.0172 (11)	0.0189 (11)	−0.0002 (8)	−0.0047 (9)	−0.0023 (9)
C14	0.0452 (19)	0.067 (2)	0.065 (2)	0.0210 (16)	0.0385 (16)	0.0228 (17)
N1A	0.005 (3)	0.029 (5)	0.038 (6)	0.001 (2)	−0.001 (3)	0.013 (4)
C16A	0.015 (2)	0.011 (4)	0.016 (4)	−0.006 (3)	−0.007 (3)	−0.002 (3)
C17A	0.025 (2)	0.027 (3)	0.033 (3)	−0.0048 (18)	0.0040 (19)	0.004 (2)
C18A	0.033 (3)	0.029 (3)	0.033 (3)	−0.018 (2)	−0.008 (2)	0.009 (2)
C19A	0.039 (4)	0.048 (4)	0.047 (4)	−0.029 (3)	−0.025 (3)	0.023 (3)
C20A	0.018 (2)	0.047 (4)	0.056 (4)	−0.010 (2)	−0.010 (2)	0.031 (3)
N1B	0.006 (4)	0.023 (5)	0.029 (5)	−0.007 (3)	−0.002 (3)	0.006 (3)
C16B	0.019 (3)	0.021 (7)	0.026 (6)	−0.010 (4)	−0.005 (4)	−0.002 (4)
C17B	0.025 (3)	0.045 (3)	0.025 (3)	−0.014 (2)	−0.004 (2)	0.010 (2)
C18B	0.023 (3)	0.042 (4)	0.041 (3)	−0.019 (2)	−0.002 (2)	0.009 (3)
C19B	0.026 (3)	0.054 (5)	0.038 (4)	−0.020 (3)	−0.011 (3)	0.016 (3)
C20B	0.033 (3)	0.030 (3)	0.028 (3)	−0.022 (2)	−0.014 (2)	0.008 (2)

### Geometric parameters (Å, º)

**Table:**

Pt1—N3^i^	2.0293 (16)	N1A—C20A	1.449 (14)
Pt1—N3	2.0293 (16)	N1A—C16A	1.49 (2)
Pt1—Cl1^i^	2.3108 (5)	C16A—C17A	1.574 (11)
Pt1—Cl1	2.3108 (5)	C16A—H16A	0.9700
N3—C2	1.307 (3)	C16A—H16B	0.9700
N3—C4	1.471 (3)	C17A—C18A	1.528 (6)
N5—C6	1.463 (3)	C17A—H17A	0.9700
N5—C4	1.481 (2)	C17A—H17B	0.9700
N5—O1	1.485 (2)	C18A—C19A	1.525 (7)
O1—C2	1.362 (2)	C18A—H18A	0.9700
O13—C10	1.372 (3)	C18A—H18B	0.9700
O13—C14	1.417 (3)	C19A—C20A	1.525 (9)
C2—N1B	1.333 (17)	C19A—H19A	0.9700
C2—N1A	1.353 (16)	C19A—H19B	0.9700
C4—C7	1.504 (3)	C20A—H20A	0.9700
C4—H4	0.9800	C20A—H20B	0.9700
C6—H6A	0.9600	N1B—C16B	1.45 (2)
C6—H6B	0.9600	N1B—C20B	1.520 (16)
C6—H6C	0.9600	C16B—C17B	1.496 (18)
C7—C8	1.386 (3)	C16B—H16C	0.9700
C7—C12	1.389 (3)	C16B—H16D	0.9700
C8—C9	1.385 (3)	C17B—C18B	1.530 (6)
C8—H8	0.9300	C17B—H17C	0.9700
C9—C10	1.383 (3)	C17B—H17D	0.9700
C9—H9	0.9300	C18B—C19B	1.544 (8)
C10—C11	1.395 (3)	C18B—H18C	0.9700
C11—C12	1.391 (3)	C18B—H18D	0.9700
C11—H11	0.9300	C19B—C20B	1.497 (10)
C12—H12	0.9300	C19B—H19C	0.9700
C14—H14A	0.9600	C19B—H19D	0.9700
C14—H14B	0.9600	C20B—H20C	0.9700
C14—H14C	0.9600	C20B—H20D	0.9700
			
N3^i^—Pt1—N3	180.0	C17A—C16A—H16A	110.2
N3^i^—Pt1—Cl1^i^	90.20 (5)	N1A—C16A—H16B	110.2
N3—Pt1—Cl1^i^	89.80 (5)	C17A—C16A—H16B	110.2
N3^i^—Pt1—Cl1	89.80 (5)	H16A—C16A—H16B	108.5
N3—Pt1—Cl1	90.20 (5)	C18A—C17A—C16A	111.2 (7)
Cl1^i^—Pt1—Cl1	180.0	C18A—C17A—H17A	109.4
C2—N3—C4	106.24 (16)	C16A—C17A—H17A	109.4
C2—N3—Pt1	133.39 (15)	C18A—C17A—H17B	109.4
C4—N3—Pt1	120.06 (12)	C16A—C17A—H17B	109.4
C6—N5—C4	113.41 (17)	H17A—C17A—H17B	108.0
C6—N5—O1	104.81 (17)	C19A—C18A—C17A	110.2 (4)
C4—N5—O1	100.75 (14)	C19A—C18A—H18A	109.6
C2—O1—N5	103.55 (16)	C17A—C18A—H18A	109.6
C10—O13—C14	117.7 (2)	C19A—C18A—H18B	109.6
N3—C2—N1B	128.4 (6)	C17A—C18A—H18B	109.6
N3—C2—N1A	133.4 (6)	H18A—C18A—H18B	108.1
N1B—C2—N1A	19.0 (6)	C20A—C19A—C18A	111.8 (4)
N3—C2—O1	114.03 (19)	C20A—C19A—H19A	109.3
N1B—C2—O1	116.7 (6)	C18A—C19A—H19A	109.3
N1A—C2—O1	111.4 (6)	C20A—C19A—H19B	109.3
N3—C4—N5	101.72 (15)	C18A—C19A—H19B	109.3
N3—C4—C7	116.68 (17)	H19A—C19A—H19B	107.9
N5—C4—C7	108.50 (16)	N1A—C20A—C19A	109.5 (7)
N3—C4—H4	109.8	N1A—C20A—H20A	109.8
N5—C4—H4	109.8	C19A—C20A—H20A	109.8
C7—C4—H4	109.8	N1A—C20A—H20B	109.8
N5—C6—H6A	109.5	C19A—C20A—H20B	109.8
N5—C6—H6B	109.5	H20A—C20A—H20B	108.2
H6A—C6—H6B	109.5	C2—N1B—C16B	128.7 (13)
N5—C6—H6C	109.5	C2—N1B—C20B	120.2 (12)
H6A—C6—H6C	109.5	C16B—N1B—C20B	111.1 (12)
H6B—C6—H6C	109.5	N1B—C16B—C17B	111.5 (12)
C8—C7—C12	119.0 (2)	N1B—C16B—H16C	109.3
C8—C7—C4	118.74 (19)	C17B—C16B—H16C	109.3
C12—C7—C4	122.15 (19)	N1B—C16B—H16D	109.3
C9—C8—C7	120.8 (2)	C17B—C16B—H16D	109.3
C9—C8—H8	119.6	H16C—C16B—H16D	108.0
C7—C8—H8	119.6	C16B—C17B—C18B	111.4 (6)
C10—C9—C8	120.0 (2)	C16B—C17B—H17C	109.4
C10—C9—H9	120.0	C18B—C17B—H17C	109.4
C8—C9—H9	120.0	C16B—C17B—H17D	109.4
O13—C10—C9	115.2 (2)	C18B—C17B—H17D	109.4
O13—C10—C11	125.0 (2)	H17C—C17B—H17D	108.0
C9—C10—C11	119.9 (2)	C17B—C18B—C19B	109.3 (4)
C12—C11—C10	119.5 (2)	C17B—C18B—H18C	109.8
C12—C11—H11	120.2	C19B—C18B—H18C	109.8
C10—C11—H11	120.2	C17B—C18B—H18D	109.8
C7—C12—C11	120.7 (2)	C19B—C18B—H18D	109.8
C7—C12—H12	119.6	H18C—C18B—H18D	108.3
C11—C12—H12	119.6	C20B—C19B—C18B	109.9 (5)
O13—C14—H14A	109.5	C20B—C19B—H19C	109.7
O13—C14—H14B	109.5	C18B—C19B—H19C	109.7
H14A—C14—H14B	109.5	C20B—C19B—H19D	109.7
O13—C14—H14C	109.5	C18B—C19B—H19D	109.7
H14A—C14—H14C	109.5	H19C—C19B—H19D	108.2
H14B—C14—H14C	109.5	C19B—C20B—N1B	111.0 (7)
C2—N1A—C20A	124.3 (11)	C19B—C20B—H20C	109.4
C2—N1A—C16A	120.9 (11)	N1B—C20B—H20C	109.4
C20A—N1A—C16A	114.6 (12)	C19B—C20B—H20D	109.4
N1A—C16A—C17A	107.4 (8)	N1B—C20B—H20D	109.4
N1A—C16A—H16A	110.2	H20C—C20B—H20D	108.0
			
N3^i^—Pt1—N3—C2	−49 (15)	C8—C9—C10—C11	−1.0 (3)
Cl1^i^—Pt1—N3—C2	116.2 (2)	O13—C10—C11—C12	−179.11 (19)
Cl1—Pt1—N3—C2	−63.8 (2)	C9—C10—C11—C12	−0.5 (3)
N3^i^—Pt1—N3—C4	138 (15)	C8—C7—C12—C11	−0.7 (3)
Cl1^i^—Pt1—N3—C4	−56.47 (15)	C4—C7—C12—C11	−177.36 (17)
Cl1—Pt1—N3—C4	123.53 (15)	C10—C11—C12—C7	1.4 (3)
C6—N5—O1—C2	150.49 (18)	N3—C2—N1A—C20A	−172.2 (6)
C4—N5—O1—C2	32.6 (2)	N1B—C2—N1A—C20A	105 (4)
C4—N3—C2—N1B	−175.5 (7)	O1—C2—N1A—C20A	−5.4 (10)
Pt1—N3—C2—N1B	11.1 (8)	N3—C2—N1A—C16A	2.7 (12)
C4—N3—C2—N1A	160.1 (6)	N1B—C2—N1A—C16A	−80 (4)
Pt1—N3—C2—N1A	−13.3 (7)	O1—C2—N1A—C16A	169.5 (7)
C4—N3—C2—O1	−6.4 (3)	C2—N1A—C16A—C17A	125.3 (10)
Pt1—N3—C2—O1	−179.78 (15)	C20A—N1A—C16A—C17A	−59.3 (12)
N5—O1—C2—N3	−17.2 (3)	N1A—C16A—C17A—C18A	55.0 (11)
N5—O1—C2—N1B	153.3 (5)	C16A—C17A—C18A—C19A	−54.4 (8)
N5—O1—C2—N1A	173.3 (5)	C17A—C18A—C19A—C20A	54.6 (8)
C2—N3—C4—N5	27.1 (2)	C2—N1A—C20A—C19A	−124.4 (8)
Pt1—N3—C4—N5	−158.46 (13)	C16A—N1A—C20A—C19A	60.3 (10)
C2—N3—C4—C7	144.93 (19)	C18A—C19A—C20A—N1A	−56.2 (9)
Pt1—N3—C4—C7	−40.6 (2)	N3—C2—N1B—C16B	−30.5 (13)
C6—N5—C4—N3	−146.99 (18)	N1A—C2—N1B—C16B	83 (4)
O1—N5—C4—N3	−35.54 (19)	O1—C2—N1B—C16B	160.6 (9)
C6—N5—C4—C7	89.4 (2)	N3—C2—N1B—C20B	153.6 (5)
O1—N5—C4—C7	−159.12 (16)	N1A—C2—N1B—C20B	−93 (4)
N3—C4—C7—C8	144.73 (18)	O1—C2—N1B—C20B	−15.2 (9)
N5—C4—C7—C8	−101.2 (2)	C2—N1B—C16B—C17B	−118.6 (11)
N3—C4—C7—C12	−38.6 (3)	C20B—N1B—C16B—C17B	57.6 (10)
N5—C4—C7—C12	75.5 (2)	N1B—C16B—C17B—C18B	−57.4 (11)
C12—C7—C8—C9	−0.8 (3)	C16B—C17B—C18B—C19B	55.5 (9)
C4—C7—C8—C9	175.95 (18)	C17B—C18B—C19B—C20B	−55.6 (7)
C7—C8—C9—C10	1.7 (3)	C18B—C19B—C20B—N1B	56.9 (9)
C14—O13—C10—C9	−176.8 (2)	C2—N1B—C20B—C19B	118.3 (8)
C14—O13—C10—C11	1.8 (3)	C16B—N1B—C20B—C19B	−58.2 (10)
C8—C9—C10—O13	177.74 (19)		

Symmetry code: (i) −*x*, −*y*+1, −*z*.

### Hydrogen-bond geometry (Å, º)

**Table:**

*D*—H···*A*	*D*—H	H···*A*	*D*···*A*	*D*—H···*A*
C14—H14*C*···Cl1^ii^	0.96	2.76	3.423 (3)	127

Symmetry code: (ii) −*x*−1/2, *y*−1/2, −*z*+1/2.
